# *Pasteurella bettyae* Infections in Men Who Have Sex with Men, France

**DOI:** 10.3201/eid3007.240352

**Published:** 2024-07

**Authors:** Andy Li, Florian Herms, Dominique Pataut, Jean-Baptiste Louison, Charles Cassius, Manel Merimèche, Jean David Bouaziz, Béatrice Berçot, Sébastien Fouéré

**Affiliations:** Hôpital Saint-Louis Centre for Genital and Sexually Transmitted Diseases, Paris, France (A. Li, F. Herms, D. Pataut, J.-B. Louison, C. Cassius, J.D. Bouaziz, S. Fouéré);; Hôpital Saint Louis National Reference Centre for Bacterial Sexually Transmitted Infections, Paris (M. Merimèche, B. Berçot);; French Institute for Medical Research (INSERM) Joint Research Unit 1137, Paris (M. Merimèche, B. Berçot)

**Keywords:** *Pasteurella bettyae*, bacteria, antimicrobial resistance, men who have sex with men, MSM, sexually transmitted infections, balanitis, balanoposthitis, France

## Abstract

*Pasteurella bettyae* is a gram-negative bacillus sporadically involved in human infections; its main reservoirs are cats and dogs. A recent publication suggests the possibility of sexual transmission leading to genital infections in men who have sex with men. We report 9 cases in France of genital infection among this population.

*Pasteurella bettyae* is a gram-negative bacillus for which main reservoirs are cats, dogs, other mammals, and birds. *P. bettyae* has caused infections of the human genitourinary tract (*1*) and lungs (*2*) and can be transmitted through neonatal sepsis (*3*). A recent publication from Spain suggested possible sexual transmission in 2 men who have sex with men (MSM) (*4*). We describe 9 cases of *P. bettyae* genital infections in MSM in France during 2018–2022. As required by national ethics regulations, all patients received written information concerning the retrospective use of anonymized data; none expressed opposition to use and publication of those data. 

We extracted clinical and biologic data from medical and bacteriology laboratory records. All 9 patients were MSM who sought care at the Hôpital Saint-Louis Center for Genital and Sexually Transmitted Diseases in Paris, France. Patients were 22–58 (mean 41.33) years of age. Seven patients were living in Paris, 1 in a northern suburb of Paris, and 1 in the region north of Paris closest to the city. The average number of sexual partners was 10.4; 2 patients were in monogamous relationships. Six patients did not use protection; 3 used condoms except for oral sex. No patients were HIV-positive, but 1 was immunosuppressed because of a kidney transplant. Two were receiving preexposure prophylaxis (PrEP) for HIV (Table 1). 

**Table 1:** Demographic and behavioral characteristics, bacteriological data, clinical manifestations, and evolution of *Pasteurella bettyae* infection among 9 men who have sex with men, France*

Pt no.	Year pos	Age	Partners/ y	Animal contact	Condom use	PrEP	Clinical manifestation	Culture site	Other pathogens retrieved	*P. bettyae* AMR	Antimicrobial treatment (follow-up)
1	2018	52	10	N	Y, exc oral	N	Balanitis	Coronal sulcus	None	None	None (healing)
2	2018	34	8	N	Y, exc oral	N	Balanitis	Coronal sulcus	*Haemophilus parainfluenzae*, *Finegoldia magna*	None	Cefixime (none)
3	2019	46	Unk	Unk	Occ	Y	Balanitis	First void urine + urethra	*H. parainfluenzae*	None	None (none)
4	2020	22	1	N	N	N	Balanoposthitis	Coronal sulcus	None	Penicillin G	None (none)
5	2020	27	10	Unk	Occ	N	Urethritis	Urethra	*Neisseria gonorrhoeae*	None	Ceftriaxone/ doxycycline (none)
6	2021	22	8	Unk	Y, exc oral	N	Balanitis	Glans penis	*Streptococcus dysgalactiae*	None	None (none)
7	2021	54	35	Cat	N	Y	Genital ulcer	Coronal sulcus	*C. trachomatis*	None	Penicillin G benzathine/ doxycycline (ulcer healing)
8	2021	56	10	Cows	Occ	N	Punctuate balanoposthitis	Coronal sulcus	*S. agalactiae*, *F. magna*	TMP/SMX	None (partial healing on zinc oxide paste)
9	2022	58	1	Unk	N	N	Persistent genital ulcer 2 wk after primary syphilis treatment	Coronal sulcus	None	Penicillin G/ ampicillin	Amoxicillin/ clavulanate (healing with indurated scar)

*AMR, antimicrobial resistance; exc, except; occ, occasional; pos, positive; PrEP, preexposure HIV prophylaxis; pt, patient; TMP/SMX, trimethoprim/sulfamethoxazole; unk, unknown.

One patient had a cat, another acknowledged contact with cows 2 weeks before clinical signs appeared, and 2 reported no contact with animals; data were missing for the other 4 patients. The main clinical manifestations were balanitis (4/9, 44.4%) and balanoposthitis (2/9, 22.2%). Balano-preputial sulcus ulcers in 2 patients were ultimately diagnosed as lymphogranuloma venereum and syphilis and urethral discharge in 1 patient was diagnosed as gonorrhea. No other signs or symptoms, including fever or lymphadenopathy, were reported (Table 1). 

According to national guidelines, we presumptively treated patients with antimicrobial drugs; those with ulcers received benzathine benzylpenicillin plus doxycycline and the patient with urethral discharge received ceftriaxone plus doxycycline. We targeted co-pathogens when detected by nucleic acid amplification tests. One patient who had not received antimicrobial drugs attended a review visit and recovered after receiving specific treatment (cefixime) (Table 1). 

We obtained *P. bettyae* colonies after 24-hour culture on polyvitex agar (bioMérieux, https://www.biomerieux.com) at <5% CO_2_ and 35°C–37°C. We identified *P. bettyae* using Vitek MS matrix-assisted laser desorption/ionization time-of-flight mass spectrometry (bioMérieux). We used disk diffusion (BioRad Laboratories, https://www.bio-rad.com) on polyvitex agar to test antimicrobial susceptibility and interpreted results according to European Committee on Antimicrobial Susceptibility Testing recommendations (*5*). All *P. bettyae* isolates showed susceptibility to amoxicillin/clavulanic acid, ceftriaxone, tetracycline, and fluoroquinolones, as is usually observed for this genus. One isolate from patient 9 exhibited resistance to amoxicillin and penicillin G (penicillin G MIC of 0.75 mg/L), 1 from patient 4 to penicillin G only, and 1 from patient 8 to trimethoprim/sulfamethoxazole (Table 1). 

We performed whole-genome sequencing and bioinformatics analysis of 6/9 available *P. bettyae* isolates (BioProject accession no. PRJNA1039245) as described elsewhere (*6*). We developed the distance matrix of *Pasteurella* clinical isolates and rooted it by comparison with the genome reference sequence of *P. bettyae* strain CCUG 2042 (National Center for Biotechnology Information Reference Sequence database accession no. NZ_AJSX01000007.1). We observed relatedness between all *P. bettyae* isolates (Table 2), highlighting that 4 isolates, from patients 1, 3, 5, and 6, were closely related, varying among them by only 12–270 single-nucleotide polymorphisms and by 587–628 SNPs from the *P. bettyae* CCUG 2042 strain described in 2012 in the United States. Isolates from patients 4 and 7 were more distant but had ribosomal identification and 16S rRNA of *P. bettyae.*

**Table 2:** Differences in single-nucleotide polymorphisms among 6 *Pasteurella bettyae* isolates from 9 men who have sex with men, France, and a reference strain*†

Isolate source	CCUG 2042	Patient 7	Patient 4	Patient 6	Patient 5	Patient 1	Patient 3
CCUG 2042	0	4,551	4,729	587	587	628	587
Patient 7	4,551	0	1,714	4,665	4,665	4,540	4,595
Patient 4	4,729	1,714	0	4,913	4,915	4,766	4,823
Patient 6	587	4,665	4,913	0	12	179	270
Patient 5	587	4,665	4,915	12	0	181	270
Patient 1	628	4,540	4,766	179	181	0	97
Patient 3	587	4,595	4,823	270	270	97	0
*Reference *P. bettyae* strain CCUG 2042 from National Center for Biotechnology Information Reference Sequence database (accession no. NZ_AJSX01000007.1). Numbers in cells indicate distances in SNPs.							

The 9 cases of genital *P. bettyae* infection exclusively in MSM we describe clustered within a 4-year period; no case was registered at our hospital before 2018. Fewer than 50 cases (mainly genital) have been reported worldwide across the previous 60 years, in male and female patients. In this series, the most specific clinical manifestation was balanitis/balanoposthitis. Because only 1 patient received targeted treatment, we could not deduce that *P. bettyae* was solely responsible for his symptoms or treatment responsible for his recovery. In case-patients with ulcers and urethritis, *P. bettyae* superinfection was more likely. Only half of patients for whom information was available had contact with animals, which provides insufficient support to determine direct anthropozoonotic transmission. Two thirds of patients reported not using condoms and the remaining third not using them for oral sex, which is not enough evidence to determine the transmission route and preventive efficacy of using condoms. However, if balanitis is indeed the main clinical manifestation, condoms provide an obvious physical barrier. Of note, the first case in this cluster occurred 2 years after PrEP policy implementation in France, but whether receding usage of condoms by PrEP users had any part in this emergence remains speculative. 

*P. bettyae* appears to be an emerging cause of sexually transmitted genital infection among MSM in Europe (*3*). More case descriptions are needed to delineate its clinical spectrum and appropriate handling. We encourage physicians to test bacterial swab samples when managing similar genital symptoms, especially balanitis. 
